# Etiology of Hunner Lesions: Linking Immune Dysregulation and Chronic Inflammation in Interstitial Cystitis/Bladder Pain Syndrome

**DOI:** 10.1007/s00192-026-06739-9

**Published:** 2026-06-20

**Authors:** Eboni T. Acoff, Kaylee Ferrara, Hannah Ruetten, Gopal Badlani, Robert J. Evans, Amr El Haraki, Stephen J. Walker

**Affiliations:** 1 Department of Urology, Wake Forest University School of Medicine, Winston-Salem, NC USA; 2 Wake Forest Institute for Regenerative Medicine, 391 Technology Way NE, Winston-Salem, NC 27101 USA

**Keywords:** Autoimmune, Biomarkers, Hunner lesions, IC/BPS, Pathophysiology

## Abstract

**Introduction and Hypothesis:**

Hunner lesions (HL) are distinctive ulcerative bladder lesions characterized by a central scar and surrounding erythema. Traditionally, HL has been used as a clinical diagnostic tool for a subset of patients with interstitial cystitis/bladder pain syndrome (IC/BPS); however, emerging evidence supports a unique pathophysiology for IC/BPS with HL (HIC) that is distinct from IC/BPS without HL (NHIC). This review is aimed at synthesizing current literature on the etiology of HL/HIC and evaluate whether HIC and NHIC warrant classification as separate clinical conditions with distinct management strategies.

**Methods:**

A comprehensive literature review using PubMed was conducted to identify literature surrounding HL etiology and pathophysiology. Case–control studies, cohort studies, experimental studies, case reports, and animal studies were included; editorial comments and literature reviews were excluded from this review.

**Results:**

Emerging data suggest that a subset of patients might be predisposed to HIC, including alteration in major histocompatibility complex (MHC) molecules, HLA amino acid positions, and additional protein expression alterations detected by RNA sequencing. Hypoxia-inducible factor activation and chronic ischemia have also been shown to play a role in HL development. Both local and systemic inflammation are seen in HIC, with clonal B-cell expansion influencing the upregulation of inflammatory cytokines, chemokines, and lymphoplasmacytic cells. With additional validation these biomarkers have the potential to serve as a diagnostic tool.

**Conclusion:**

Hunner lesion with IC/BPS is a chronic condition likely caused by an upregulation of humoral immunity leading to an overexpression of inflammatory markers within the urothelium, suggesting an autoimmune-mediated pathology for this patient subgroup.

## Introduction

Hunner lesions (HLs), historically referred to as Hunner ulcers, are not true ulcers but rather distinctive bladder lesions characterized by a central scar and surrounding erythema, detected upon cystoscopy [1]. First described in 1915 by American gynecologist, Guy Leroy Hunner [1], these lesions occur in a subset of patients suffering from chronic, seemingly idiopathic, bladder pain. If HLs are observed, they are considered a specific diagnostic marker for interstitial cystitis/bladder pain syndrome (IC/BPS). However, only a small subset of patients diagnosed with IC/BPS have identifiable HLs on cystoscopy, whereas the overwhelming majority do not. Further, cystoscopy is not currently required for an IC/BPS diagnosis and therefore many patients are never evaluated for HLs.

In the USA, IC/BPS is diagnosed as a single overarching condition of bladder discomfort that has a heterogenous clinical presentation. However, diagnostic criteria and terminology differ around the globe and emerging evidence aggregated and presented at the Global Consensus meeting on IC/BPS in 2025 suggests that IC/BPS with HLs (HIC) might differ fundamentally from IC/BPS without HLs (NHIC) in pathophysiology, clinical presentation, and treatment response [2–4]. In fact, the European Society for the Study of Interstitial Cystitis (ESSIC) specifically subclassifies IC/BPS with HLs as the “BPS/IC ESSIC Type 3C” subgroup [5]. This review is aimed at summarizing the current literature on the etiology of HIC and determining whether there is sufficient evidence to classify HIC and NHIC as separate conditions.

## Materials and Methods

**Fig. 1:**
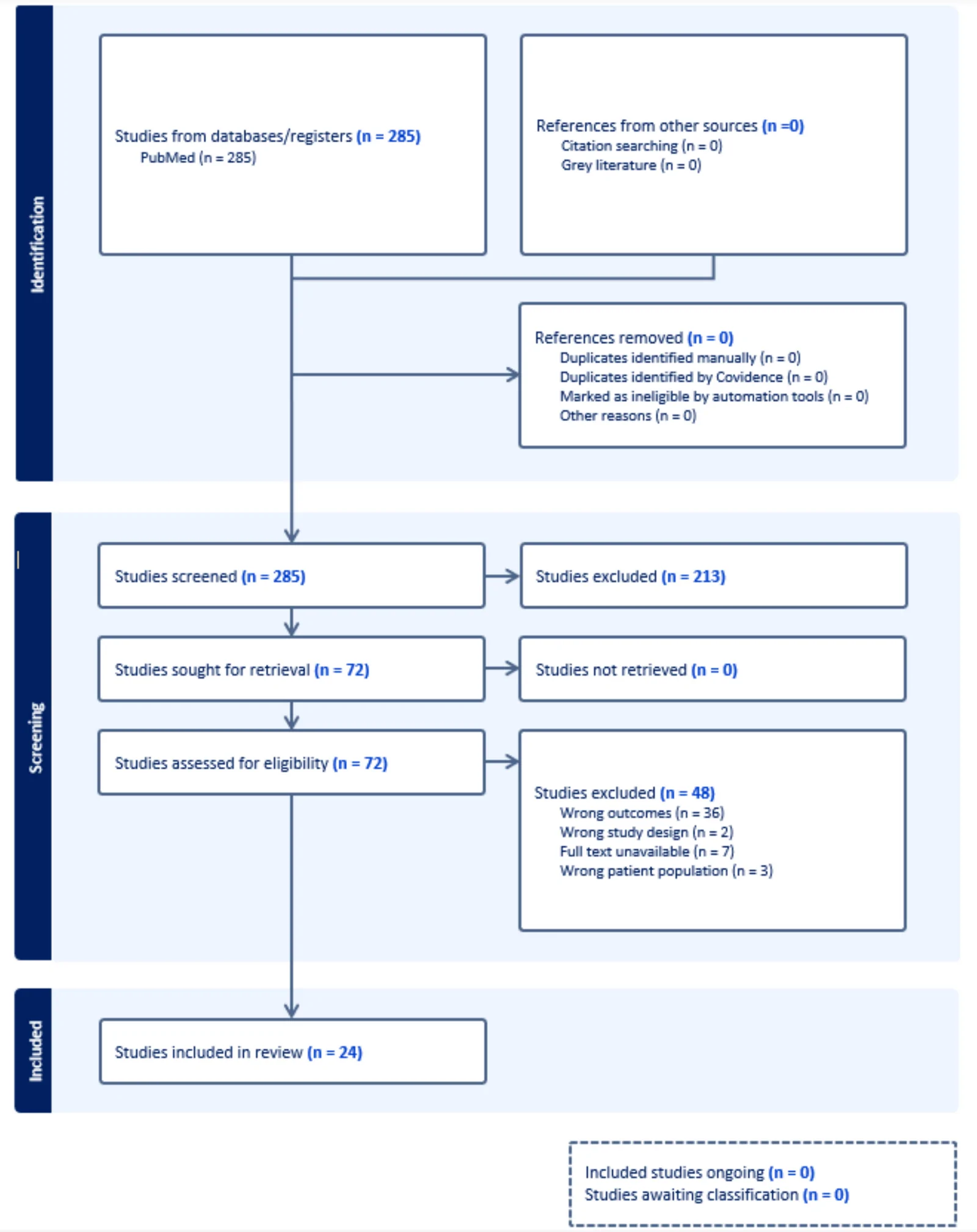
Preferred Reporting Items for Systematic Reviews and Meta-Analyses diagram

A comprehensive literature review was conducted using PubMed with the following search terms: (hunner lesion OR hunner’s OR HL OR hunner’s lesion OR hunner’s ulcer OR BPS type 3 OR ESSIC type 3 C) AND (bladder pain syndrome OR painful bladder syndrome OR interstitial cystitis OR IC/BPS) AND (etiology OR pathophysiology OR cause OR origin OR pathology). Covidence systematic review software was used to aid study organization and manuscript screening [6]. Case–control studies, cohort studies, experimental studies, case reports, and animal studies were included; editorial comments and literature reviews were excluded. Publication countries included the USA, Japan, Taiwan, Sweden, and China, with publication dates ranging from 1989 to 2024. A total of 213 titles and abstracts were screened for inclusion. Upon full-text review, 24 manuscripts met the inclusion criteria (Fig. 1); these are summarized in Table 1.

**Table 1:** Primary findings of the manuscripts included

References	Level of evidence^a^	Study design	Population	Primary findings	Limitations
Akiyama et al. [7]	IV	Case–control	27 HIC patients; 15 non-IC controls	The mean sum of proportion of double-positive cells (CXCR3+ and CD138+) was significantly higher in HIC than in non-IC cystitis cases	Control samples may not be optimal. Potential sampling bias. Co-expression of chemokine markers was not confirmed by flow cytometry
Akiyama et al. [8]	IV	Case–control	33 IC/BPS patients (12 with HIC); 9 healthy controls	Overexpression of VEGF and BAFF at the RNA and protein levels was detected with an associated higher density of subepithelial microvasculature and inflammatory infiltrate in HIC bladder tissue	Small sample size. Urothelium and lamina propria were analyzed simultaneously among samples. Microvessel competency was not assessed
Akiyama et al. [9]	IV	Cohort	25 HIC patients who underwent electrocautery of HL with HOD	109 genes were differentially expressed between HL and nonlesion areas. 74 genes were up-regulated and 35 were down-regulated in HL. Upregulated genes were associated with anatomical development, signal transduction, plasma membrane development, and enzymatic binding	Small sample size. Result interpretation is limited owing to a lack of validation using independent resources. Upregulation of HIF-1α is not identified as an etiology vs a consequence of inflammation
Akiyama et al. [10]	IV	Case–control	144 HIC patients; 41,516 controls	A completely linked haplotype of Thr71-Glu74-Leu75 showed a protective effect against HIC. HLA-DQβ1 and HLA-DPβ1 variants were associated with increased odds of HIC	Sampling bias. Recruited patients must have presented with typical clinical and histological features of HIC and controls were not specifically confirmed to be free of IC/BPS. Potentially low generalizability owing to only including participants of Japanese ancestry
Akiyama et al. [11]	IV	Case–control	25 HIC patients; 13 BCG cystitis controls	Patients with HIC had worse symptom severity scores when compared with patients with BCG cystitis. The down-regulated DEGs associated with HIC were related to biological processes, biosynthetic processes, and cellular components	Small sample size. Results were not validated using independent resources. Microbial transcriptomes in the RNA-sequencing analysis were not investigated
Enerbäck et al. [12]	IV	Case–control	16 HIC patients; 15 NHIC controls	All washings contained mast cells, but mast-cell number and histamine content were more closely related to symptom severity and cystoscopic findings	Small sample size. All patients were women
Horie et al. [13]	IV	Case–control	Flash-frozen: 37 HIC patients; 14 BCG cystitis controls; 5 chronic cystitis controls; 5 follicular cystitis controls	Temporal and spatial expansion of B cells in HIC bladders was identified using BCR repertoire analysis with next-generation RNA sequencing. B cell clones were found within the entire HIC-affected bladder	Small sample size. BCR repertoire analysis was only performed of the IgG clones. The mechanism of APRIL/BAFF signaling has not been studied in vivo
			Formalin: 13 HIC patients		
Ichihara et al. [14]	IV	Case–control	Human: 10 HIC patients; 8 bladder cancer controls	Stronger expression of TLR7 was seen in both urothelial cells and inflammatory cells of HIC samples compared with controls. There was no difference in TLR7 expression between HL and nonlesion areas of HIC biopsies	TLR7 expression was not examined in pathologies other than HIC. The effects of TLR7 agonist were not examined past 96 h in mouse bladders. The association of TLR7 with chronic inflammation or nerve fibers was not explored
		Animal model	Animal: C57BL/6N female mice	Mucosal TLR7 expression and inflammatory cells significantly increased in mouse bladders 96 h after loxoribine instillation (a TLR7 agonist). Post-instillation, pain-like licking behavior and voiding frequency increased whereas voiding volume decreased	
Jhang et al. [15]	IV	Case–control	490 IC/BPS patients undergoing HOD with at least grade 1 glomerulations	E-cadherin, CK14, CK20, P63, and FGFR3/4 levels were significantly lower in HIC biopsies than in NHIC biopsies. CK5 and BAX expression were increased in HIC urothelium	Limited sample size of biopsies. Bladder specimens were obtained by random biopsy and may not be representative of the whole bladder. Controls were taken from patients with stress urinary incontinence and may not represent a normal bladder
			Biopsies: 85 IC/BPS patients; 22 controls		
Jhang et al. [16]	IV	Case–control	Humans: 21 HIC patients; 1 NHIC control; 2 bladder cancer controls	62% of HIC biopsies were positive for EBERs. All BRLF1+ bladders were positive for LMP1 and EBNA1, indicating concurrence of both latent and lytic EBV infection	Small sample size. Lack of clinical evidence to support anti-EBV drugs in treating HIC. Only female rats were used
		Animal model	Animals: 30 female Sprague–Dawley rats divided into five subgroups	LCL caused severe adhesion of the bladder to perivesicular fat 14 days after implantation and induced more redness and edema than lytic LCL in rat bladders. An erythematous lesion with neovascularization and a central scar was observed in the bladders of the LCL and lytic LCL groups. BDNF expression was increased among these bladders when compared with the control group	
Kadekawa et al. [17]	III	Animal model	66 female adult Sprague–Dawley rats divided into two groups: EAC (*n* = 38); sham-treated (*n* = 14)	EAC rates showed increased frequency of urination and decreased voided volume, along with an irregular surface of umbrella cells and uneven staining of the urothelium when compared with controls. EAC rats showed upregulation of CXCL1, CXCL10, CXCL11, and NGF	The animal model uses an induced IC/BPS-like cystitis so there is limited applicability to humans. Only female rats were used
Lee et al. [18]	IV	Case–control	68 HIC patients; 40 NHIC controls; 34 healthy controls	A significant difference in βD-2 expression was shown between HIC and both the control and NHIC groups. The number of mast cells correlated positively with βD-2 concentration within tissues	Retrospective, single study center has susceptibility to selection bias and relies on the accuracy of records. Mast-cell and βD-2 levels may be affected by comorbidities. Mast-cell count was not assessed in the control group. Urine-based studies are difficult in IC/BPS patients owing to limited bladder capacity and low biomass of urine
Logadottir et al. [19]	IV	Case–control	10 HIC patients; 4 NHIC controls	HIC specimen showed strong immunoreactivity and nitrotyrosine expression within the urothelium. These findings strengthen the notion of an up-regulation of iNOS in HIC as opposed to a macrophage-based pathology	Small sample size
Logadottir et al. [20]	IV	Case–control	12 HIC patients; 6 healthy controls	Significantly increased mRNA expression of IL-17A, IL-10, and IL-6 in HIC patients. TNF-α, TGF-β, and IFN-γ mRNA levels did not differ	Small sample size. Controls were taken from patients with stress urinary incontinence and may not represent a normal bladder
Logadottir et al. [21]	IV	Cohort	379 IC/BPS patients; healthy controls	HIC specimen had higher levels of nitric oxide and showed evidence of chronic inflammation within the bladder mucosa. IL-6, IL-17, and IL-10 expressions were significantly higher in HIC when compared with controls	Unknown number and lack of description of the control group
Lu et al. [22]	IV	Cross-sectional	9 IC/BPS with normal bladder capacity; 4 IC/BPS with low bladder capacity (2 HIC); 3 healthy controls	HIC tissues had increased CD4 + memory T cell activation and decreased resting mast cell and activated NK cell activation. The results suggest that HIC signaling might be like that of immune rejection and autoimmune diseases	Small sample size. Lack of verification of biological samples
Lyu et al. [23]	IV	Case–control	153 HIC patients; 46,087 controls	Three amino acid positions in HLA-DQβ1 contributing to MHC class II molecule antigen presentation were associated with increased HIC risk. Several autoimmune diseases revealed statistical significance in the bidirectional MR analysis with HIC	Lack of GWAS data of non-HIC subtypes of IC/BPS limit the ability to investigate and differentiate the genetic associations between subtypes and immune-related disorders. Environmental and other extrinsic factors must be considered with investigating risk factors for the occurrence of HIC and autoimmune diseases
Maeda et al. [24]	IV	Case–control	27 HIC patients; 39 NHIC controls; 26 non-IC cystitis controls	Most HIC specimens revealed inflammatory change with a denuded epithelium compared with NHIC specimens, which were infrequently inflamed with preserved epithelium. Although affiliated with pancystitis, the extent of inflammation and denudation was more severe in HL than in nonlesion areas. Clonal B cell expansion was observed in over 30% of HIC cases, but often found in only one of the two biopsies	Small sample size. The utility of histological evaluation in HIC vs NHIC diagnosis is limited
Moldwin et al. [25]	IV	Case–control	Biopsies: 48 HIC patients; 2 benign bladder tumor controls	There was a more than ×50 increase in CD138+ plasma cells in HIC samples compared with controls. CD20+ B cells, TNF, and IL-6 concentrations were also elevated among HIC specimens	Small sample size. Study design cannot determine whether the presence of B cells is a consequence or a cause of disease. Biopsy samples and urine samples were conducted asynchronously so correlation analysis could not be performed
			Urine samples: 18 HIC patients; 18 NHIC controls; 4 unaffected controls		
Peng et al. [26]	IV	Case–control	HIC patients; healthy controls	Findings significant for a decrease in UPK3A + urothelial cells and decreased urothelial thickness in HIC. There was an upregulation of pro-inflammatory genes (e.g., TNF and IL-6) as well as downregulation of KRT20 and ZO-1, suggesting a malfunction of the urothelial barrier or damage to cells responsible for its formation	Unknown sample size. Controls were taken from patients with stress urinary incontinence and may not represent a normal bladder
Pontari et al. [27]	IV	Case–control	43 IC/BPS patients; 30 healthy controls	Bladder blood flow decreased with filling among IC/BPS patients and increased among controls. Basal blood flow was not different across groups. Perfusion decreased with HOD in IC/BPS patients and increased with HOD in controls	Small sample size. Perfusion could not be measured in a continuous manner so intravesical pressure at which perfusion decreased could not be determined
Scheffler et al. [28]	VI	Case–report	73-year-old female with cystocele, entero-rectocele, HIC, glomerulations, and low bladder capacity (300 ml)	After uterosacral ligament repair, the patient experienced decreased frequency of voiding and nocturia, as well as complete resolution of HL and increased bladder capacity of 700 ml. This supports the theory that bladder pain may arise from unmyelinated c-fibers owing to the inability of muscle forces to tense the bladder base enough to support the stretch receptors secondary to loose suspensory ligaments	Study design limits the generalizability of the results to larger populations. Suggested etiology of HIC would not explain HL presentation in male or young female patients
Tseng et al. [29]	IV	Case–control	5 HIC patients; 5 healthy controls	27 distinct genes showed a substantial -fold increase in bladder epithelial cells from both HL and nonlesion areas. Among these, two high-affinity receptors of IgG and IgE, and four cytokine/chemokine receptors were up-regulated	Small sample size. The data derived from GeneChips is subject to both experimental and analytical errors. The results of this study are relatively preliminary
Ward et al. [30]	IV	Case–control	4 HIC patients; 4 NHIC controls	34 proteins were significantly increased in diseased HIC tissues compared with diseased NHIC tissues, 10 of which are involved in the immune response. 5 proteins involved in collagen formation and cellular adhesion were decreased in HIC tissues compared with NHIC tissue. Diseased HIC tissue showed a downregulation of proteins involved in the ubiquitination pathway	Small sample size. Only female patients were included. Lack of healthy controls

*BDNF* brain-derived neurotrophic factor, *BCG* Bacillus Calmette–Guérin, *DEGs* differentially expressed genes, *EAC* experimental autoimmune cystitis, *EBV* Epstein–Barr virus, *GWAS* genome-wide association studies, *HIC* IC/BPS with HL, *HL* Hunner lesion, *HOD* hydrodistension, *IC/BPS* interstitial cystitis/bladder pain syndrome, *LCL* lymphoblastoid cell line, *NHIC* IC/BPS without HL

^a^Level of evidence reflects the quality and strength of each manuscript. I: systematic reviews or meta-analyses of randomized control trials; II: randomized control trials; III: controlled trials without randomization (quasi-experimental studies; IV: non-experimental studies; V/VI: systematic reviews of descriptive or qualitative studies; VII: opinion pieces, committee reports, literature reviews

## Etiology of Interstitial Cystitis/Bladder Pain Syndrome with Hunner Lesion

It is important to first acknowledge that the identification of HIC proves to be both difficult and inconsistent as it requires a cystoscopy, and a certain level of skill and knowledge on the part of the surgeon to accurately identify HL; therefore, the true prevalence of HL within the IC/BPS population is unknown, with estimates ranging from 4–56% of patients [31]. In all the included studies cystoscopy was performed to identify HL. If HIC is to be classified as a distinct subtype, additional training would need to take place to ensure that all urologists diagnosing IC/BPS patients can accurately identify HL, and cystoscopy ought to be implemented in the diagnostic process.

**Fig. 2:**
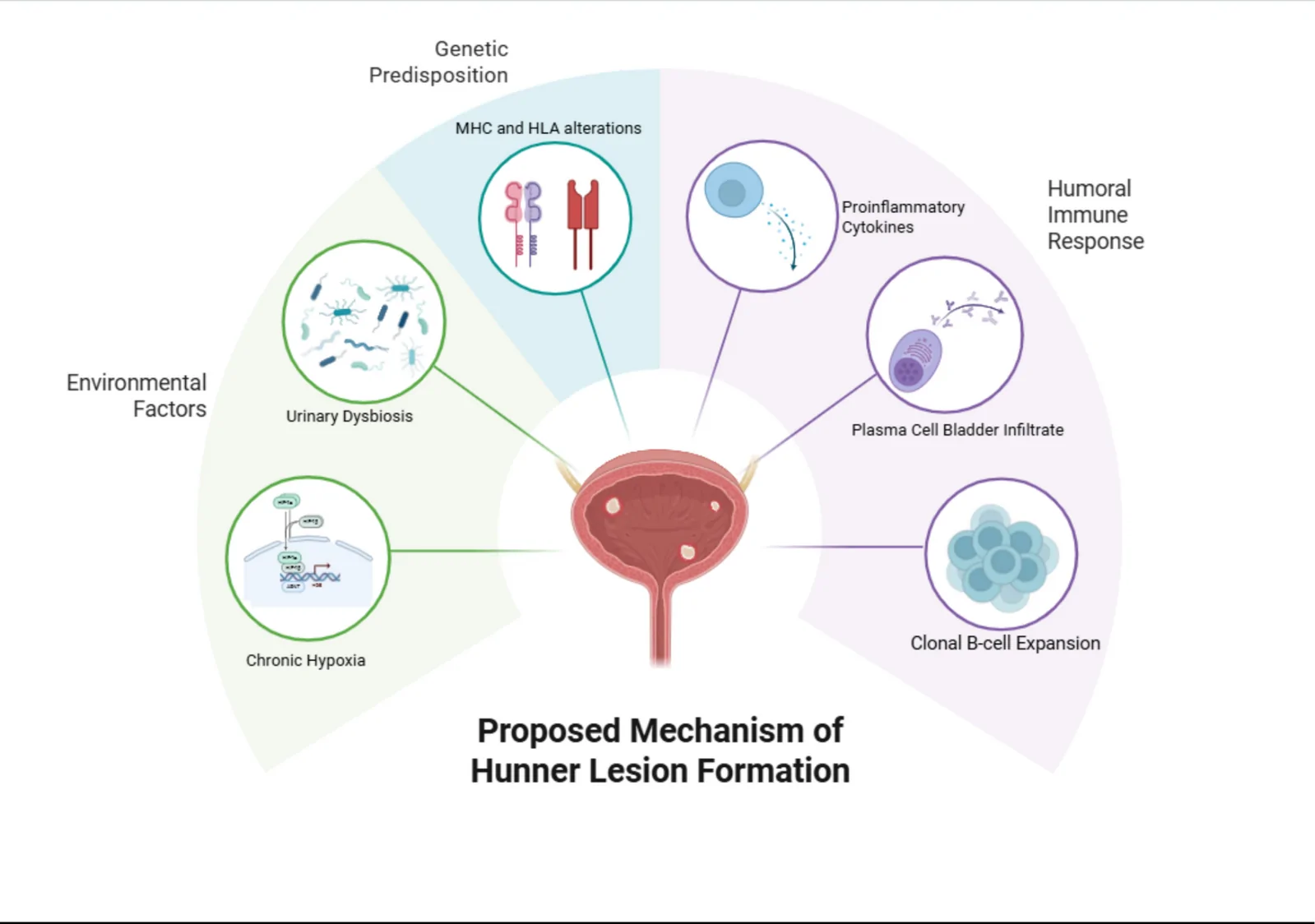
Pathophysiological hypothesis to development of interstitial cystitis/bladder pain syndrome with Hunner lesion

As with most other conditions with a similar post-pubescent onset and variable symptomology, it is likely that the development of these lesions is multifactorial, requiring an unfortunate conglomeration of circumstances that lead to HIC (Fig. 2). Nevertheless, there is a mounting body of evidence that HIC patients suffer from a unique, highly inflammatory, potentially autoimmune disorder with hypoxia playing a role in lesion development [3, 4, 31].

### Local Versus Systemic Inflammation

It is well known that HIC is associated with an increase in inflammatory cells within the urothelium; however, it is unclear whether this inflammation leads to bladder injury or occurs secondary to bladder injury. Nevertheless, the inflammatory response is multivariate, with evidence of B cell, T cell, and plasma cell infiltrates, affirmed by the presence of CD20, CD3, CD4, and CD138 on immunohistochemistry (IHC) staining of HIC bladder biopsies [16, 22, 25].

Patients with HIC have altered gene expression in multiple pathways, with alterations appearing both systemically and locally within the bladder. Tseng et al. analyzed bladder epithelial samples from patients with HIC versus healthy controls and identified a gene expression signature within the urothelium for which 40 genes were upregulated in samples taken directly from HL [29]. Analysis of transcriptional responses revealed a potential molecular mechanism involving upregulation of major histocompatibility complex (MHC) class I and II molecules, IgG- and IgE-mediated mast-cell activation, and upregulation of T cell antigen receptors [29]. Considering that MHC molecules play a significant role in antigen presentation and processing, these findings suggest that patients could have a predisposition to autoimmune-mediated lesion formation in the bladder.

Tseng et al. also identified an upregulation of various cytokines and chemokines (i.e., IL2RB, IL10RA, CMKLR1) throughout the bladder, not just within HL, suggesting that inflammation might not be confined to the lesion areas but is widespread throughout the entire HIC bladder [29]. An additional study of biopsies from HL regions of HIC bladders demonstrated a higher concentration of plasma cells than the nondiseased areas within the same bladders [22]. Increased expression of Toll-like receptor (TLR7) in both urothelial cells and inflammatory cells within the lamina propria has also been associated with the development of HIC, with upregulation noted within lesion sites and in surrounding bladder tissue [14].

There appear to be profound differences in protein expression within HIC bladders when comparing biopsies taken within the lesion itself with those taken from the “normal” or nondiseased areas (NDAs). HL areas of HIC bladders exhibit marked downregulation of the ubiquitination pathway, which is primarily involved in marking cells for destruction, compared with adjacent NDAs. Furthermore, NDA of HIC tissues had significant downregulation of 4 out of 50 proteins involved in the Rap1 cell adhesion signaling pathway (RALA, PFN2, GNAI1, and CTNNB1) when compared with NDA tissues of NHIC bladder samples [30], underscoring whole-bladder molecular differences beyond localized lesions. The alteration of these pathways suggests that the urothelial cells in areas where HL occur might be less likely to be marked for destruction after cell damage, making these tissues more susceptible to long-term scarring as opposed to the restorative processes of degradation and regeneration.

These findings suggest an increased severity of whole-bladder inflammation in HIC compared with NHIC or normal bladder and even further increases in inflammation in HL areas compared with NDAs.

### Mast Cells and Dysbiosis

The role of mast cells in IC/BPS pathophysiology remains ambiguous, as some research has shown a significant increase in mast-cell concentration within IC/BPS whereas other studies show no significant difference in concentration when compared with control groups. Coincidentally, mast-cell presence and concentration appear to be related more to symptom severity than disease development [12]. Notably, mast-cell concentration appears to correlate positively with β-defensin 2 (βD-2), an antimicrobial peptide, which has been shown to have a significantly higher presence in urine samples from HIC patients than in NHIC and control groups [18]. The presence of βD-2 may suggest that the chronic inflammation observed in HIC could be caused by urinary dysbiosis, an imbalance of beneficial and harmful bacteria leading to loss of the normal microbial diversity within the bladder, creating an environment of chronic bladder inflammation [12, 18]. IC/BPS diagnostic criteria require that bladder pain occur in the absence of infection or other identifiable cause; however, a history of previous infections and/or non-infectious over-colonization of bladder microbiota is a plausible proposed mechanism for HIC and warrants further exploration.

### Interstitial Cystitis/Bladder Pain Syndrome with Hunner Lesion as an Autoimmune Condition

Clonal B cell expansion is a process related to the adaptive immune response, causing increased synthesis of B cells designed to target a specific antigen. Many biopsies from HIC bladders have shown an increase in lymphoplasmacytic cells, epithelial denudation, and an overall increase in stromal inflammation, which has been attributed to clonal B cell expansion [13, 24, 30]. Jhang et al. reported the presence of latent EBV-infected B cells within HIC tissues; these B cells were subsequently shown to have upregulation of brain-derived neurotrophic factor (BDNF) expression, which the authors proposed as a contributing factor in HIC pathogenesis [16]. BDNF expression is known to play a role in nerve hyperplasia and hyperalgesia seen in irritable bowel syndrome, so it is hypothesized that this increased expression contributes to the bladder pain and nerve hyperplasia seen in IC/BPS [16]. Another study attributed B cell expansion to the presence of APRIL/BAFF upregulation seen in HIC [13]. These cytokines play a crucial role in B cell survival, maturation, and antibody production and have been significantly associated with multiple autoimmune diseases. Additionally, T follicular helper (Tfh) cells, which are involved in autoimmune processes and B cell-mediated pathologies, exhibit increased expression in HIC tissues compared with normal controls [22]. In comparison, most NHIC bladders do not show evidence of stromal inflammation [24]. These findings provide evidence for an autoimmune component in the etiology of HIC.

### Genetic Predisposition

Although there is limited evidence to substantiate that IC/BPS is a heritable condition, emerging data suggest that a subset of patients have genetic differences that predispose them to HIC. Two genome-wide association studies identified MHC regions as a susceptibility locus for HIC [10, 23]. Researchers found multiple variants in human leukocyte antigen (HLA) amino acid positions, which were associated with an increased risk of HIC, particularly HLA-DQβ1 and HLA-DPβ1, which play a role in antigen presentation among MHC class II molecules [10, 23]. Notably a methionine-to-leucine substitution at amino acid position 178 of the HLA-DPβ1 conferred an odds ratio of 2.35, whereas a linked haplotype of HLA-DQβ1 in positions 71, 74, and 75 conferred an odds ratio of 1.94 [10, 23]. The individual diversity of HLA amino acids plays a significant role in immune-cell activation and has been studied extensively in relation to autoimmune disease risk, transplant rejection, and vaccination development. This evidence suggests that variations in both MHC and HLA genotypes might be associated with an increased risk for the development of HIC.

### Hypoxia-Induced Bladder Injury

In addition to a potential autoimmune origin, evidence suggests that HL formation might occur as a secondary response to local hypoxia, wherein oxidative damage to the urothelium is caused by reactive oxygen species, a downstream byproduct of hypoxia-inducible factor (HIF-1) activation in low oxygen conditions. HIF-1α protein has been shown to be upregulated in HIC bladders with significantly higher concentration in HL areas when compared with NDA, indicating that ischemia likely contributes to HL development [9]. RNA sequencing from bladder mucosal biopsy tissues has shown an increase in the expression of VEGF and BAFF; this overexpression was found to be significantly related to symptom severity in patients with HIC when compared with patients with NHIC, patients with hypersensitive bladder (HSB), and control patients [8]. Inducible nitric oxide synthase (iNOS) also appears to play a specific role in the development of HIC, as iNOS activity is minimal in NHIC tissues [19, 20].

When considering the anatomy of the bladder, it is important to account for changes in size, position, and blood flow to the organ at different times during the micturition cycle. In both healthy and diseased bladders, blood flow to the trigone and superficial layer of the bladder body tends to decrease during bladder filling; however, in patients with IC/BPS, reperfusion after emptying is significantly slower in the deeper tissue layers than in the superficial layer in the same patients and compared with the entire bladder in healthy controls [27]. Therefore, it is unclear from this study whether decreased perfusion is associated with the development of IC/BPS or if the perfusion deficit is reactive to the changes in bladder physiology associated with IC/BPS. Nevertheless, repeated exposure to a hypoxic environment could be an inciting factor or contribute to disease progression in HIC.

One example in support of this theory is a case report that describes a 73-year-old woman with a history of HIC and pelvic multi-organ prolapse. The report suggests that the patient might have “cured” of the disorder after surgical apical repair with tissue fixation system (TFS) tensioned tape [28]. Prior to surgery, the patient had a bladder capacity of 430 ml with co-occurring urgency, patchy mucosal reddening, and HL seen in the bladder during hydrodistension. Interestingly, her postoperative bladder capacity increased to 700 ml, with complete resolution of urinary symptoms and previously identified bladder lesions after undergoing repeat hydrodistension. Investigators hypothesize that the resolution of her symptomology was secondary to the TFS providing additional support to the uterosacral ligaments (USLs), stating that USL laxity leads to chronic mucosal venous outflow obstruction and chronic hypoxia of bladder tissues. This chronic hypoxia then induces a local inflammatory response, leading to an HIC phenotypical presentation [28]. This case report, in addition to the previous literature linking HIF-1 overexpression to HL, supports the hypothesis that chronic hypoxia of bladder tissues may play a significant role in the underlying pathophysiology of HL.

## Interstitial Cystitis/Bladder Pain Syndrome with Hunner Lesion Biomarkers as a Diagnostic Tool

Interstitial cystitis/bladder pain syndrome is often a diagnosis of exclusion; therefore, increasing interest in developing diagnostic tools to aid in early identification and treatment of the disorder remains. In this regard, various inflammatory biomarkers have been associated with IC/BPS, with few showing promising specificity for the HIC phenotype. Chemokines such as CXCR3 and CD138 have been shown to be significantly increased in HIC cases, compared with decreased levels of E-cadherin, CK14, and CK20 within HIC urothelium [7, 15, 25]. Multiple cytokines have also been identified as potential biomarkers for HIC, including IL-17, IL-10, and IL-6 [17, 20, 21, 26]. One study identified a downregulation of UPK3A + urothelial cells in HIC bladders that correlated with decreased urothelial thickness and malfunction of the urothelial barrier [26]. Researchers presumed that the cell damage was mediated by TLR3 and NR2F6, which trigger programmed urothelial cell destruction, leading to the development of ulcers [26]. Although the use of biomarkers as diagnostic tools for IC/BPS remains relatively underdeveloped, identification and validation of these specific genes and proteins may yield useful targets not only for diagnosis and treatment but also for understanding the mechanisms that underlie HL development.

## Animal Model Studies

A notable gap in IC/BPS research is the limited availability of relevant animal models to study etiology. Although emerging technologies—such as organoid-based research—and the limited use of animals with naturally occurring IC-like syndromes (i.e., cats) have shifted much of the field away from traditional animal models, there remains value in investigating experimentally induced IC-like syndromes in animals. Such models may help to delineate key differences in pathological features that are difficult to capture through alternative approaches. For example, one study that induced experimental autoimmune cystitis (EAC) in rats using bladder-tissue homogenate from donor rat bladder tissue [17] found that resultant bladder specimens had an increase in inflammatory markers (CXCL1, CXCL10, CXCL11, and NGF) as well as having evidence of irregular umbrella cell surface and disruption of the basal epithelium [17]. Another study hypothesized that TH1/17 overexpression increased plasma-cell infiltration in bladders. To test this hypothesis, researchers developed an anti-mouse IFN-γ DNA aptamer to instill in experimental murine models and found that inflammation, voiding dysfunction, and pelvic nociception were significantly improved in their study cohort [11]. These two studies highlight the potential benefit that animal-based research can have toward not only identifying etiology but also testing potential therapeutic agents.

## Limitations

This study was conducted via a literature search discussing the etiological mechanisms of HL; however, it is not without limitations. First, IC/BPS is a condition with variable presentation and therefore does not have a specific diagnostic criterion. Because of this, many patients may be mis-diagnosed, leading to potential gaps in research among some populations. Our group only utilized one research database in this study (PubMed) and thus may have missed any articles not available through this resource. Additionally, the diagnosis of HIC is dependent on clinical identification of HL on cystoscopy, which can be variable based on physician experience, patient factors, and the potential waxing and waning status of these lesions. Many studies included in this review had a small sample size, with the largest single-study sample consisting of 153 HIC patients. The small sample sizes in conjunction with the lack of longitudinal data make it difficult to both generalize this study’s findings to the entire HIC population and comment on disease progression. Although animal studies have the potential to show causation through controlled experiments, most of the evidence provided in this review can only support statements of association. Further research is needed to reinforce the hypotheses currently identified within the published literature.

## Conclusions

Interstitial cystitis/bladder pain syndrome with Hunner lesion is a chronic condition likely caused by an upregulation of humoral immunity leading to an overexpression of inflammatory markers within the urothelium, suggestive of an autoimmune-mediated pathology. The stimulus leading to this robust inflammatory response is likely a combination of genetic and environmental factors, including mutations and upregulation of MHC proteins and HLA amino acid substitutions, chronic hypoxia leading to HIF-1α overexpression, overexpression of vascular growth factors such as VEGF and BAFF, and urinary dysbiosis. There is minimal evidence to support a similar immune response in NHIC bladders, supporting the hypothesis that HIC and NHIC have distinct etiological backgrounds. Biomarkers (e.g., CXCR3, IL-17, and TLR3) could prove a useful tool for both diagnostic and therapeutic targets, but further research is needed to identify specific cytokines, growth factors, or other proteins that can be identified from serum, urine, or tissue samples. The findings of this review are limited by the paucity of published research available on this subject; however, the evidence that was able to be gleaned from published work provides strong support for an autoimmune-based etiology. Although definitive confirmation of the underlying pathophysiology of HIC cannot be made without further primary investigation, the current evidence provides a compelling rationale for the classification of HIC and NHIC as distinct IC/BPS subtypes.
